# Aesthetic recovery of alveolar atrophy following autogenous onlay bone grafting using interconnected porous hydroxyapatite ceramics (IP-CHA) and resorbable poly-L-lactic/polyglycolic acid screws: case report

**DOI:** 10.1186/1472-6831-14-60

**Published:** 2014-06-02

**Authors:** Kazumi Kubozono, Masaaki Takechi, Kouji Ohta, Shigehiro Ono, Takayuki Nakagawa, Shinichi Fujimoto, Nobuyuki Kamata

**Affiliations:** 1 Department of Oral & Maxillofacial Surgery, Graduate School of Biomedical & Health Sciences, Hiroshima University, 1-2-3 Kasumi, Minami-Ku, Hiroshima 734-8553, Japan

**Keywords:** Autogenous onlay bone grafting, Interconnected porous hydroxyapatite ceramics (IP-CHA), Bioresorbable poly-L-lactic/polyglycolic acid (PLLA-PGA) screw

## Abstract

**Background:**

Onlay bone grafting techniques have some problems related to the limited volume of autogenous grafted bone and need for surgery to remove bone fixing screws. Here, we report a case of horizontal alveolar ridge atrophy following resection of a maxillary bone cyst, in which autogenous onlay bone grafting with interconnected porous hydroxyapatite ceramics (IP-CHA) and bioresorbable poly-L-lactic/polyglycolic acid (PLLA-PGA) screws was utilized.

**Case presentation:**

A 51-year-old man had aesthetic complications related to alveolar atrophy following maxillary bone cyst extraction. We performed onlay grafting for aesthetic alveolar bone recovery using IP-CHA to provide adequate horizontal bone volume and PLLA-PGA screws for bone fixing to avoid later damage to host bone during surgical removal. During the operation, an autogenous cortical bone block was collected from the ramus mandibular and fixed to the alveolar ridge with PLLA-PGA screws, then the gap between the bone block and recipient bone was filled with a granular type of IP-CHA. Post-surgery orthopantomograph and CT scan findings showed no abnormal resorption of the grafted bone, and increased radiopacity, which indicated new bone formation in the area implanted with IP-CHA.

**Conclusion:**

Our results show that IP-CHA and resorbable PLLA-PGA screws are useful materials for autogenous onlay bone grafting.

## Background

Alveolar atrophy after removal of a tumor or cyst leads to unfavorably proportioned prosthetic treatment, frequently resulting in shapeless crowns, or bridges with artificially created gingiva and missing papillae. Autogenous onlay bone grafting techniques for prosthetic treatment are used extensively in such cases to restore bone volume by laying autogenous bone directly onto the surface of the host bone [1,2]. However, there are problems related to the limited thickness of autogenous cortical bone and resorption of grafted bone during healing.

Recently, interconnected porous hydroxyapatite ceramics (IP-CHA) materials with high porosity have been developed and used successfully in the field of orthopedics [3,4]. IP-CHA granules consist of a porous sintered body made of hydroxyapatite ceramics and have a unique pore structure, thus they undergo extensive incorporation into host bone more rapidly than conventional interconnected porous hydroxyapatite ceramics (C-CHA) [5,6]. IP-CHA may also be useful as a bone substitute to add greater thickness to the alveolar ridge in onlay grafting augmentation for recovery of alveolar atrophy.

An autogenous bone block is generally fixed with titanium screws to the recipient bone site in onlay grafting treatment, as such screws provide rigid fixation with the block. However, autogenous bone can be affected by mechanical stress and has a risk of infection related to the removal operation. In addition, titanium screw fixation has been shown to produce radiographic artifacts [7-9]. Therefore, resorbable screws are useful alternatives, as no removal is needed.

We previously reported successful clinical results of maxillary sinus floor augmentation with mixed grafts composed of cortical bone and IP-CHA granules [10]. Application of IP-CHA and resorbable screws for onlay bone grafting may provide advantages by minimizing the need for an additional surgical procedure. Here, we report a case of severe horizontal alveolar ridge atrophy after extraction of a maxillary bone cyst in which autogenous onlay bone grafting using IP-CHA and bioresorbable poly-L-lactic/polyglycolic acid (PLLA-PGA) screws were used to restore an adequate alveolar bone volume and eliminate the need for surgery to remove bone fixing screws.

## Case presentation

**Figure 1:**
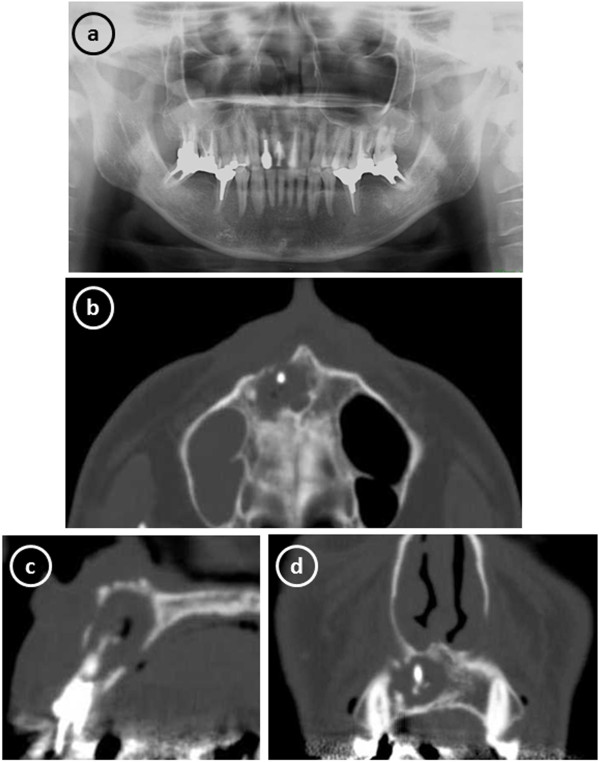
**Radiographic images obtained at the initial examination showing a periapical cystic lesion associated with the maxillary right central incisor. (a)** Orthopantomograph. **(b)** axial reconstruction, **(c)** sagittal reconstruction, and **(d)** coronal reconstruction of computed tomography scans.

A 51-year-old man was referred to our hospital with a chief complaint of swelling in the anterior portion of the right maxillary region on March 2010. There was no notable medical history. Orthopantomograph and computed tomography (CT) scan findings revealed a periapical cystic lesion associated with the maxillary right central incisor (Figure 1), after which we made a diagnosis of a periapical cyst in the maxillary central incisor. The cyst and right central incisor were extracted under general anesthesia in May, and histological findings led to a diagnosis of radicular cyst.

**Figure 2:**
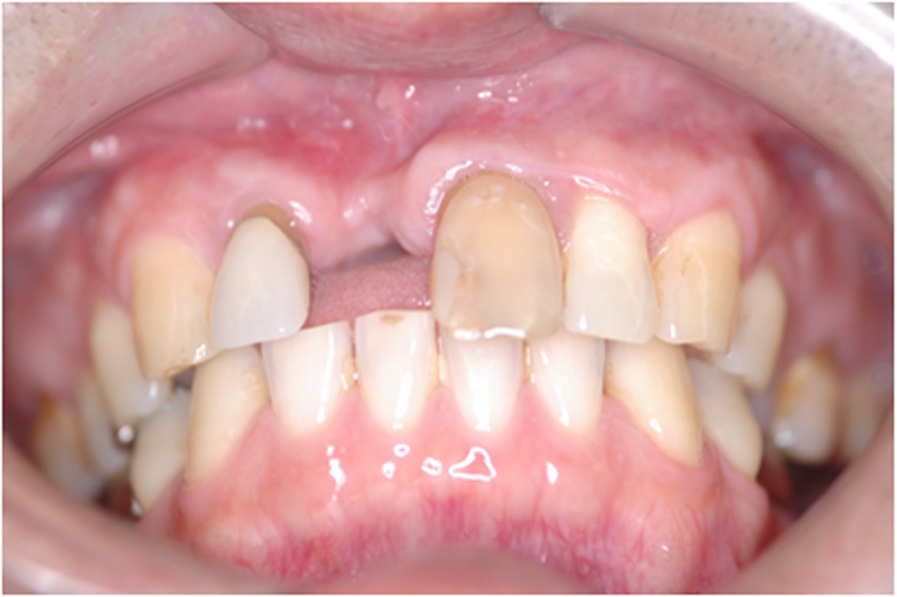
Intraoral view at 4 months after extraction of maxillary periapical cyst showing aesthetic improvement of alveolar atrophy.

**Figure 3:**
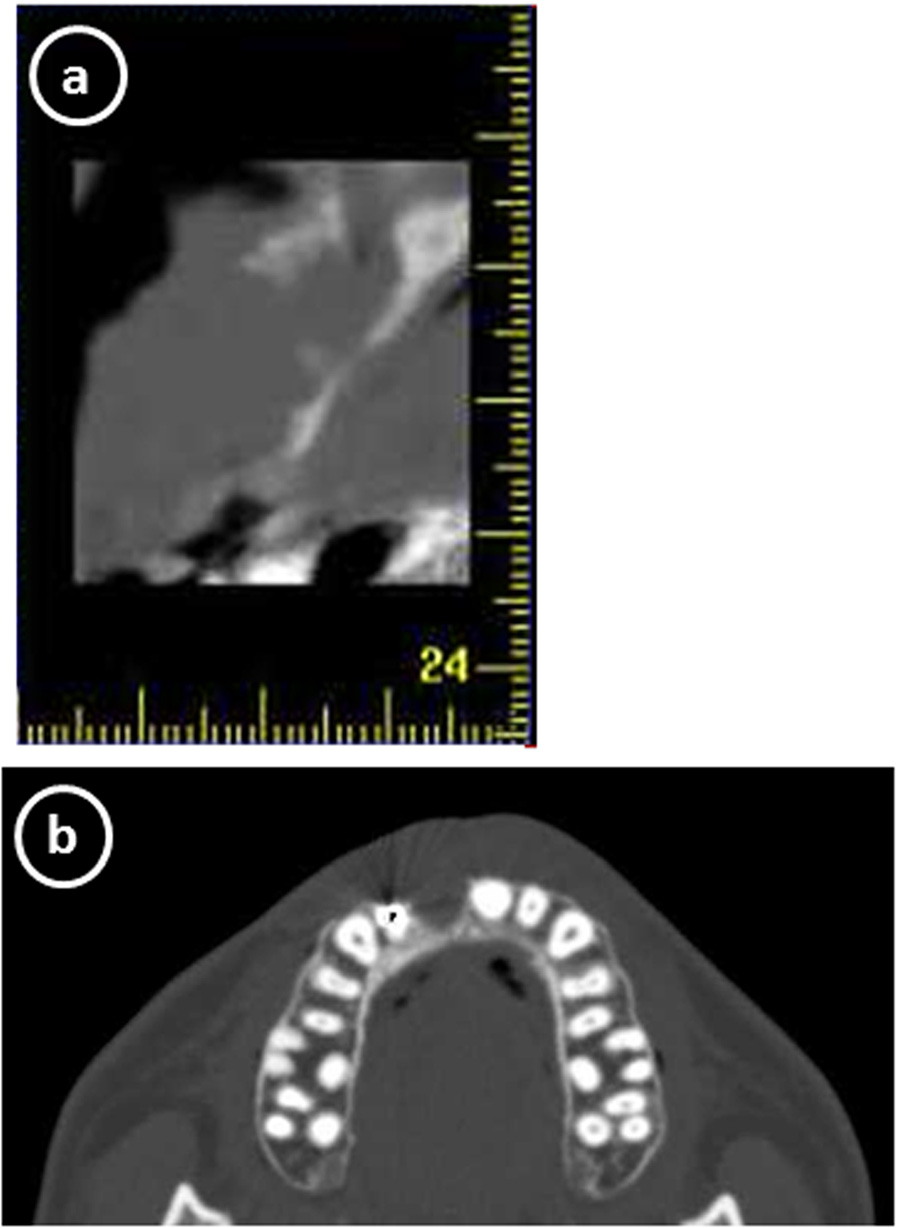
**CT scan at 4 months after extraction of maxillary bone cyst showing severe horizontal alveolar bone atrophy. (a)** Sagittal reconstruction, **(b)** axial reconstruction.

Following surgery, aesthetic complications developed (Figure 2), which CT scan images obtained 4 months after the cystectomy showed the cause to be severe horizontal alveolar bone atrophy (Figure 3). We planned to perform autogenous onlay bone grafting to restore bone volume in the alveolar ridge. It was determined that a horizontal bone volume of at least 7 mm was needed for aesthetic recovery and the width of a cortical bone block alone would be unsatisfactory to obtain an adequate alveolar ridge horizontal thickness. Therefore, we decided to use IP-CHA to fill the gap between the autogenous block bone and recipient bone, along with bioresorbable PLLA-PGA screws to fix the bone block at the recipient site.

**Figure 4:**
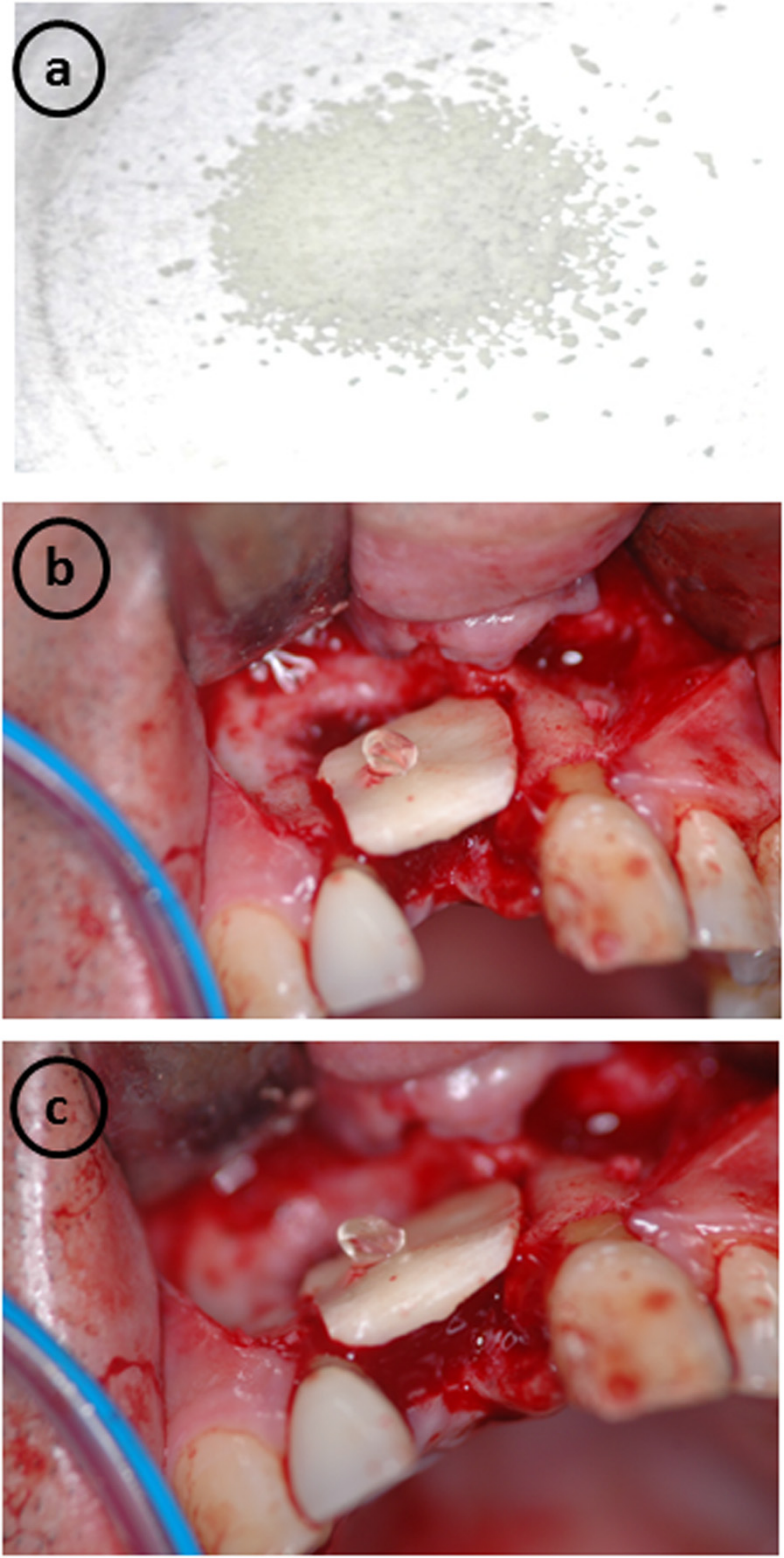
**Onlay bone grafting augmentation. (a)** Granular type-IP-CHA. **(b, c)** Fixation of autogenous bone to recipient bone using bioresorbable screws.

In October 2010, we operated on the patient under sedation and local anesthesia. To create the recipient site, a crestal incision and 2 vertical releasing incisions were made, after which a flap was raised. Next, an incision was made medial to the external oblique ridge in an anterior direction and terminated in the first molar area. Following soft tissue flap elevation, a block of bone (10 × 15 mm) was harvested for use as onlay grafting from the anterior border of the right mandibular ramus using a small round bar and fissure bur, with the sharp edges rounded off with a round bur. Gaps around the block graft were filled with a granular-type of IP-CHA (1.0-0.5 mm) (NEOBONE®, MMT, Osaka, JAPAN) and the block bone was fixed to the recipient site using resorbable screws (2 × 13 mm) (Lactosorb®, Walter Lorenz Surgical Inc., Jacksonville, FL, USA) (Figure 4). Once the graft was adapted to the site, an incision through the periosteum at the base of the flap allowed the tissue to cover the graft without tension. The recipient and donor site areas were then sutured.

**Figure 5:**
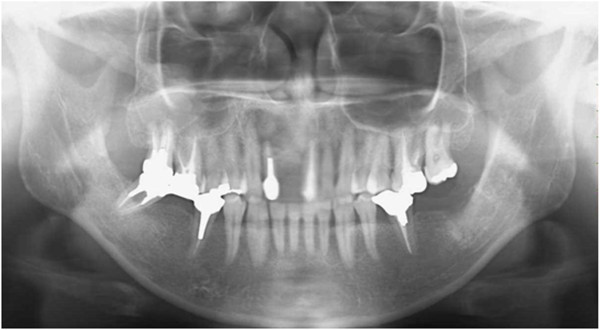
Orthopantomograph at 3 months after bone augmentation showing an increase in radiopacity, indicating new bone formation in the area implanted with IP-CHA.

**Figure 6:**
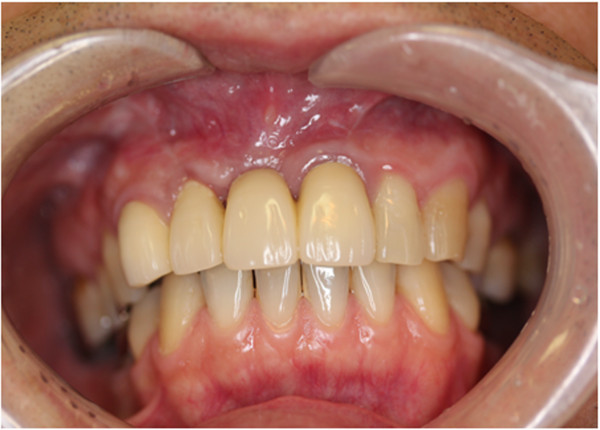
Intraoral view at 9 months after bone augmentation showing set of resin-faced facing bridges.

**Figure 7:**
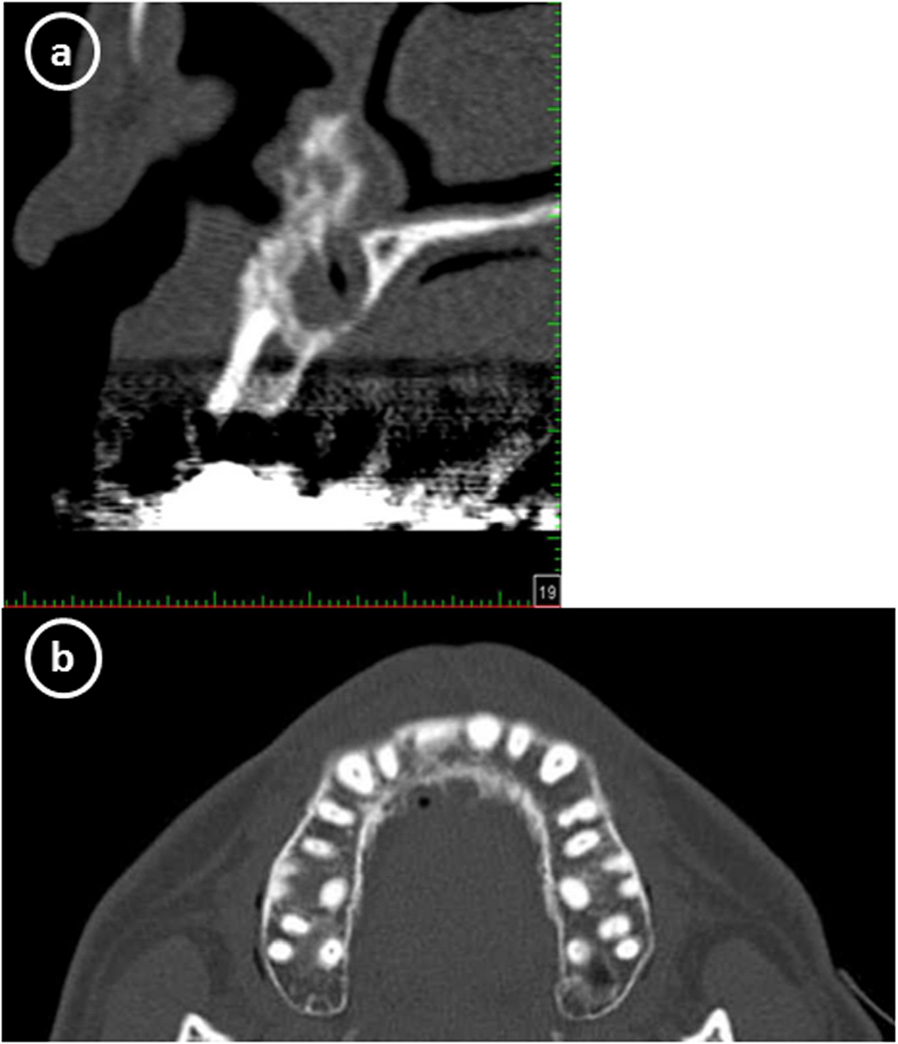
**CT scan at 15 months after bone augmentation showing no abnormal resorption of grafted bone. (a)** Sagittal reconstruction, **(b)** axial reconstruction.

An orthopantomograph obtained at 3 months after the operation showed an increase in radiopacity, indicating new bone formation in the lesion implanted with IP-CHA (Figure 5). Next, resin faced bridges were set at 9 months after the operation (Figure 6). CT scanning at 15 months after bone augmentation showed no abnormal resorption of the grafted bone (Figure 7), while at 34 months no signs of inflammation were observed in the grafted bone or IP-CHA fixed with the PLLA-PGA screws. The patient was satisfied with both the functional and aesthetic outcomes.

Titanium screws are often used to successfully achieve rigid fixation in cases with autogenous bone grafting. However, they have several potential disadvantages, such as requirement for an incision with additional soft-tissue stripping needed for removal, risk of fracture of the screws when removing, radiographic artifacts, allergic reactions, discomfort in oral mucosa, and possible restricted growth of the craniofacial skeleton in pediatric patients. As an alternative, bioresorbable fixation devices for oral and maxillofacial surgery have been well documented [7-9].

Research of bioresorbable materials has been undertaken since the early 1970s [11], with various homopolymer agents investigated including polylactic acid (PLA), polyglycolic acid (PGA), polydioxanone, and PLA and PGA copolymers [12-15]. More recently, resorbable fixation materials composed of blends of PLLA and PGA with little or no crystallinity have been reported. Such lack of crystallinity is an important factor that may allow polymer combination materials to be more easily degraded and metabolically eliminated through natural body processes, without causing inflammation or foreign body reactions [16]. In a previous study, PLLA-PGA fixation devices were completely resorbed in maxillary and mandibular bone sites without osteolysis from 18 to 24 months after surgery [17]. Such resorbable fixation materials offer many advantages for osteosynthesis over titanium screws in oral maxillofacial surgery, because several of the above-mentioned problems arising from the need to remove the bone fixing screws are eliminated [7-9]. Therefore, we used resorbable PLLA-PGA screws in the present case along with IP-CHA to avoid damage and infection of host bone caused by removal surgery.

Chacon et al. were the first to investigate a resorbable fixation system for autologous onlay bone grafting in an animal model [9]. Their study showed that resorbable screws provide excellent graft stability at the graft placement and graft retrieval stages, with no statistically significant difference between the titanium and resorbable groups in regard to the thickness of the mandibular graft site. In a clinical study, Quereshy et al. investigated the efficacy of resorbable fixation screws to fix autologous cortical onlay grafts to augment alveolar bone height and/or width in 11 patients [7]. Their results showed that cortical onlay graft integration and survivability were similar between resorbable and titanium screw fixation. In our case, no signs of inflammation or marked absorption of block bone were observed in the area of grafted bone and IP-CHA fixed with the PLLA-PGA screws. Thus, we consider that bioresorbable PLLA-PGA has no influence on graft integration, or survivability of autogenous bone and IP-CHA.

Handling of an absorbable screw may be difficult as compared to fixation with a titanium screw, as a crack in the tread of the absorbable type can occur with use of excessive force. In the present case, we utilized an absorbable screw with a hex head [18,19]. This type of head automatically separates from the screw when an appropriate amount of torque is reached. If required, the screw can be further tightened using a driver with a torque limiting system. This type of screw can be used for secure retention and to prevent damage to the screw from over tightening because of the hardness of the recipient bone, and does not require special surgeon skill.

C-CHA has been used in both craniofacial and dental applications [20,21]. However, there are few reports of C-CHA becoming fully filled by newly formed bone, which may be due to its structure and the limited connectivity between pores [22]. Inter-pore connections that are less than 2–3 μm in diameter do not allow for cell migration or vascularization into pores, events that are essential for new bone formation [5]. Thus, IP-CHA, a second generation porous calcium hydroxyapatite, was developed by use of a "form-gel" technique. This agent has a three-dimensional structure with spherical pores of uniform size interconnected by window-like holes that have diameters greater than 10 μm [6]. A previous *in vivo* study using a rabbit model reported mature bone ingrowth in IP-CHA inserted into bone defects in all of the pores [5]. Clinically, IP-CHA has been used in the fields of orthopedic surgery, including cases with osteonecrosis of the femoral condyle, juxta-articular intraosseous lesions related to rheumatoid arthritis, and benign bone tumors [6]. Our group previously performed implant placement and maxillary sinus floor augmentation with mixed grafts composed of cortical bone and IP-CHA granules in a female patient, and reported clinical behavior and histological aspects [10]. On the other hand, there are problems regarding resorption of the autogenous grafted bone during healing. In a histologic evaluation of hydroxyapatite as an onlay bone graft substitute in clinical cases over a period of 9 years, hydroxyapatite graft particles showed no signs of active resorption [23]. In the present case, we used IP-CHA to fill the gaps between autogenous and recipient bone, and bone volume in the alveolar ridge was restored after 34 months without active bone resorption. IP-CHA may be an effective bone substitute to obtain greater thickness of the alveolar ridge in autogenous onlay grafting for recovery of severe alveolar atrophy.

This is our first use of bioresorbable PLLA-PGA screws and IP-CHA during onlay grafting as a part of prosthetic treatment. No signs of postoperative infection or abnormal resorption of grafted bone were observed at 34 months after bone augmentation. Furthermore, those had no influence on graft integration and survivability of the autogenous onlay grafts. Therefore, we consider that IP-CHA and PLLA-PGA screws are useful materials for autogenous onlay bone grafting.

## Conclusion

We successfully performed onlay autogenous bone grafting using IP-CHA and resorbable PLLA-PGA screws, which were shown to be useful materials. Clinical application of this technique may have advantages including minimizing the need for an additional surgical procedure.

### Consent

Written informed consent was obtained from the patient for publication of this Case report and any accompanying images.

## Competing interests

The authors declare that they have no competing interests.

## Authors’ contributions

MT and SO performed surgery. KK, KO, and MT drafted the manuscript. TN and SF treated the patient and helped to draft the manuscript. NK conducted a review of literature and helped to draft the manuscript. All authors read and approved the final manuscript.

## Pre-publication history

The pre-publication history for this paper can be accessed here:

http://www.biomedcentral.com/1472-6831/14/60/prepub
